# Gynæcology

**Published:** 1888-08

**Authors:** S. W. Cohen

**Affiliations:** Waco, Texas


### GYNÆCOLOGY.

S. W. COHEN, WACO, TEXAS.

(Read before the Texas State Homœopathic Association, May 3d and 4th, 1888.)

Evolution is an acknowledged fact. The intelligence of the nineteenth century has ceased to argue the question. It begins with the germinal birth, so to say, or initial life of everything, be it a highly organized sentient being or a lowly shrub or flower; be it the imagination of an art or a science, call it what you may—mechanics, music, or medicine—and it follows the thread of its original purpose through every phase and change, from the inception to the highest type of its fullness. Evolution has plied her plastic hand to that special department of medicine termed gynæcology, as well as to other classical sciences. Classical! for the science was born at even date with, if not before, the time when the lyres of ancient Greece and Rome were attuned to poetry and song. Its birthplace was probably ancient Egypt, and much of gynæcological import incorporated in the *Talmud* was either borrowed from the Pharaohs or adopted from the Babylonians during the Jewish captivity. In earlier days its methods were necessarily crude, and without the kindly influence of anæsthesia, heart-rendingly cruel.

These remarks apply only to the severer operative interferences indulged in by gynæcological surgeons before the days of Chloroform or Ether. Notwithstanding these efficient pain destroyers are now in the hands of every surgeon, the spirit of evolution has done much to humanely modify gynæcological practice, and the true apostle of the healing art has become conservative in his treatment. Many crude ideas, fastened upon the medical man per *force of authoritative dictum*, have been eliminated by *force of common sense* riding abreast of prevailing theories and dogmatic methods. Many a savage and torturous surgical interference has been compelled to give way to milder and better means by a higher intelligence and the cultivated spirit of the hour. Especially has this been the case in our own school, though, judging from recent gynæcological literature of the orthodox party, or at least a progressive arm of that body, we are being closely pushed for the supremacy in this department as based on therapeutic measures. Certainly, armed with a scientific therapy, founded upon similia, we ought, *we can* have more gratifying results than the gentlemen of the antique school. The gynæcological lessons taught in some so-called homœopathic institutions are such as might have been considered choice treatment by the pupils of Hippocrates. A specular insertion, a spectacular display, and a speculative treatment, form an alliterative trio brought into requisition with but few exceptions in every gynæcological clinic. A fiery caustic, occasionally of Nitrate of Silver, then, again, of Potassa Fusa, Chromic Acid, or an admixture of Potassa Iodide and Chloral, frequently applied, without much reference to the patient's condition if the uterus appeared to be in some abnormal state, and at intervals the use of the knife was the *scientific homœopathic* (?) treatment that often held me spellbound in my college days, while my heart beat high with the hope that I, too, some day would become competent to administer *such* treatment. Internal treatment was of the most commonplace, routine order. Conium and Sepia alternately covered half the cases presented. In a few instances, perhaps, these drugs were prescribed singly; in still fewer Pulsatilla was recommended. Perhaps three or four more will cover all suggested in the gynæcological study, and all these prescribed, too, empirically, without any indication, only that this range of drugs had benefited similar conditions. At odd times the professor would patronizingly inquire, " What will we give here?" The reply, and most frequently from some first course student—a first course student, remember, is always a shrewd prescriber—would be (we'll say) " Nux." "All right," responds our professor, " give her Nux."

Thanks to my own determination, but no less thanks to the *genuine* homœopaths of our school who shed their brilliant intellect over the whole homœopathic field, bringing the kernel of truth out of every hard-shelled doubt, I have learned a better way. I haven't cauterized a cervix (for ulcerations that were never there) for several years. I haven't set up an acute perimetritis or a chronic metritis for the same length of time. I haven't used the knife to deplete a congested uterus for just as long a time. If I have rather underdone my duty than overdone it my conscience approves. I have not outraged kind nature. I have maltreated no one.

There is more quackery, more charlatanism, more deception and fraud practiced in this specialty than in any other department of medical science. Rest, simple abdominal supports, hygienic care, and the merest tampon of cotton or linen to support a heavy uterus when indicated, with proper homœopathic medication, has done more for me than severer measures. Cauterization is vicious, and viciousness is usually the legitimate offspring of ignorance. Insufflation with various pulverized preparations is less severe than other measures above referred to, but too often only expectant. Pessaries, no doubt, at times become utilitarian, but the pessary that one specialist lauds to the skies his brother practitioner decries; the methods indorsed by one are discountenanced by another. If you must have topical applications in your armamentarium, have such as are postively harmless.

Hamamelis, Calendula, and Hydrastis are known to the whole homœopathic profession. They are soothing to an inflamed mucous membrane, and this being their function, they may possibly be helpful; they can certainly not be harmful. I am aware of the fact that some cases require surgical interference, but the major portion of these find their way to the hospital. In private practice I will venture to say that four-fifths of all cases will be cured, and the other fifth ameliorated, by the properly indicated homœopathic remedy given in not too low a potency and not too often.

